# Correlation of Edema/Tumor Index With Histopathological Outcomes According to the WHO Classification of Cranial Tumors

**DOI:** 10.7759/cureus.72942

**Published:** 2024-11-03

**Authors:** Gervith Reyes Soto, Daniel Alejandro Vega-Moreno, Carlos Catillo-Rangel, Alberto González-Aguilar, Oswaldo Alan Chávez-Martínez, Vladimir Nikolenko, Renat Nurmukhametov, Andreina Rosario Rosario, Ulises García-González, Alfonso Arellano-Mata, Mario Antonio Furcal Aybar, Manuel de Jesus Encarnacion Ramirez

**Affiliations:** 1 Neurosurgical Oncology, Mexico National Cancer Institute, Tlalpan, MEX; 2 Neurosciences Unit, National Cancer Institute, Mexico City, MEX; 3 Neurosurgery, Service of the 1° de Octubre Hospital, Instituto de Seguridad y Servicios Sociales de los Trabajadores del Estado (ISSSTE), Mexico City, MEX; 4 Neurology and Psychiatry, Instituto Nacional de Ciencias Médicas y Nutrición Salvador Zubirán, Mexico City, MEX; 5 Human Anatomy and Histology, I.M. Sechenov First Moscow State Medical University (Sechenov University), Moscow, RUS; 6 Neurosurgery, Russian People's Friendship University, Moscow, RUS; 7 Medicine, Autonomous University of Santo Domingo (UASD), Santo Domingo, DOM; 8 Neurosurgery, Hospital Central Sur de Alta Especialidad "PEMEX", Mexico City, MEX; 9 Oncological Surgery, Rosa Emilia Sánchez Pérez de Tavares National Cancer Institute (INCART), Santo Domingo, DOM

**Keywords:** edema, histopathological, neurosurgery, tumor, who

## Abstract

Background: Metastatic brain tumors are a prevalent challenge in neurosurgery, with vasogenic edema being a significant consequence of these lesions. Despite the critical role of peritumoral edema in prognosis and patient outcomes, few studies have quantified its diagnostic and prognostic implications. This study aims to evaluate the correlation between the edema/tumor index (ETI) and histopathological outcomes according to the 2021 WHO classification of cranial tumors.

Methodology: We conducted a retrospective analysis of Digital Imaging and Communications in Medicine (DICOM)-format magnetic resonance imaging (MRI) data from May 2023 to May 2024, applying manual 3D volumetric segmentation using Image Tool Kit-SNAP (ITK-SNAP, version 3.8.0, University of Pennsylvania) software. The ETI was calculated by dividing the volume of peritumoral edema by the tumor volume. The study included 60 patients, and statistical analyses were performed to assess the correlation between ETI and tumor histopathology, including Receiver Operating Characteristic (ROC) curve analysis for cutoff points.

Results: A total of 60 patients were included in the study, with 27 males (45%) and 33 females (55%). The average tumor volume measured by 3D segmentation was 46.9 cubic centimeters (cc) (standard deviation [SD] ± 25.6), and the average peritumoral edema volume was 79 cc (SD ± 37.5) for malignant tumors. The ETI was calculated for each case. Malignant tumors (WHO grades 3 and 4) had a mean ETI of 1.6 (SD ± 1.2), while non-malignant tumors (WHO grades 1 and 2) had a mean ETI of 1.2 (SD ± 1.1), but this difference was not statistically significant (*P* = 0.51). ROC curve analysis for the ETI did not provide a reliable cutoff point for predicting tumor malignancy (area under the curve [AUC] = 0.59, *P* = 0.20). Despite the larger edema volume observed in malignant tumors, the ETI did not correlate significantly with the histopathological grade.

Conclusions: This study found no significant correlation between the ETI and the histopathological grade of brain tumors according to the 2021 WHO classification. While malignant tumors were associated with larger volumes of both tumor and peritumoral edema, the ETI did not prove to be a reliable predictor of tumor malignancy. Therefore, the ETI should not be used as a standalone metric for determining tumor aggressiveness or guiding clinical decision-making. Further studies with larger cohorts are required to better understand the potential prognostic value of the ETI in brain tumors.

## Introduction

Central nervous system tumors represent one of the most prevalent neurosurgical pathologies. Of these, the estimated frequency is 33% gliomas, 24% pituitary adenomas, 22% meningiomas, 6% schwannomas, and 4% others [1,2]. The edema produced by tumor lesions is considered vasogenic, characterized by a specific vascular pattern perpetuated by increased vascular permeability. Estimates of the edema/tumor correlation have been made to understand the risk of recurrence, histopathological outcome, or malignancy grade. However, previous measurements are considered *inaccurate* due to the irregularity of vasogenic edema and the significant limitations of traditional uniplanar imaging methods [3-5].

Volumetric estimates to date have mostly relied on planar methods considering lesions as perfect spheres. The advantage of manual segmentation over other volumetric methods lies in its ability to delineate each edge of a lesion using multiple slices and sequences, making it the *Gold Standard* for volume measurement [6]. In contrast, geometric volumetry is never exact, making 3D segmentation volumetry superior for volume measurement. Therefore, we resort to manual 3D volumetric segmentation whenever an exact total volume is needed [7].

Surgeons must be familiar with surgical anatomy; however, reviewing planimetric images involves certain difficulties. This tool provides surgeons with a better understanding of anatomy and anticipation of scenarios and variations during surgery, as well as near-exact volumetric measurement [8].

Automatic segmentation and volumetric methods have shown good performance and reproducibility [9]. However, manual segmentation facilitates anatomical learning during the delineation of normal and pathological structures. While automatic segmentation tools are useful for saving time, they focus only on tumor, vascular, or bone tissue, excluding areas that would be more challenging to highlight without human intervention. With manual segmentation and reconstruction, we can rebuild all these structures, expanding our 3D vision and anatomical knowledge [10].

Geometric volumetric methods approximate the volume of intracranial lesions using the principal axes in planimetric sections; however, these lesions are not spherical but rather irregular, leading to essential measurement errors. 3D volumetry has been considered a support in many fields of neurosurgery, including prognosis, function, treatment, and surgical planning [11,12].

The diagnosis of brain tumors through advanced imaging studies such as thin-section tomography or magnetic resonance imaging (MRI) is considered a challenge due to the wide distribution and frequency of these tumors and their heterogeneous characteristics [13]. Although attempts have been made to describe growth patterns with specific imaging characteristics to approach a definitive diagnosis, no imaging study is currently 100% accurate for this prediction. Therefore, we aim to describe the volumes of the tumor and its generated edema as accurately as possible to predict the histological behavior of the tumors [14].

The edema/tumor index (ETI) has been reported as an approximate, reproducible, and quantitative measure to predict the behavior of certain intracranial lesions, especially brain tumors. However, this measurement has so far been carried out in two dimensions using conventional imaging studies on two axial planes and sometimes a third coronal plane [15,16]. Cerebral edema is irregular, thus losing the volumetric principle [17].

Aims and Objectives: The primary aim of this study was to investigate whether the ETI, calculated by dividing the volume of peritumoral edema by the volume of the tumor, correlates with the histopathological grade of cranial tumors based on the 2021 WHO classification. Specifically, the objectives were to:

Quantify the volume of both tumors and peritumoral edema using advanced 3D segmentation techniques.

Calculate the ETI for each patient and compare it across different tumor grades (WHO grades 1-4).

Determine if the ETI could serve as a reliable predictor of tumor malignancy or aggressiveness.

Establish potential cutoff points for the ETI using ROC curve analysis to aid in clinical decision-making.

## Materials and methods

Study design and setting

This retrospective study was conducted by analyzing MRI studies from two tertiary care centers in Mexico: the National Institute of Neurology and Neurosurgery and the Hospital General de México Instituto Oncológico INCAN. MRI studies were initially performed on patients with a suspected diagnosis of brain tumors, and the data was reviewed in Digital Imaging and Communications in Medicine (DICOM) format for further analysis.

Inclusion criteria

The eligibility criteria for participation in the study were as follows: adult patients aged 18 years or older with a confirmed diagnosis of brain tumors classified according to the 2021 WHO guidelines. Complete MRI imaging data in DICOM format were required, including T2, T2 Fluid-Attenuated Inversion Recovery (T2 FLAIR), and contrast-enhanced T1 sequences. Additionally, complete electronic medical records must be available, containing relevant sociodemographic information.

Exclusion criteria

The following criteria were established to determine exclusion from the study: patients with incomplete or poor-quality MRI data were excluded, as were those without a definitive histopathological diagnosis. Additionally, patients with recurrent brain tumors, those who had undergone previous brain surgery or treatment, and pediatric patients under 18 years of age were also excluded from the study.

Data collection

Imaging data in DICOM format were collected from the participating institutions, including T2, T2 FLAIR, and contrast-enhanced T1 sequences. The corresponding histopathological diagnoses, including the degree of malignancy, were determined according to the 2021 WHO classification. Sociodemographic information was retrieved from the electronic medical records of each institution.

Imaging analysis

Tumor volumes were calculated using two approaches. First, 2D planimetric tumor volume calculations were performed by following the Response Evaluation Criteria in Solid Tumors (RECIST) guidelines [18,19]. The largest axial slice along the rostro-dorsal axis, parallel to the posterior commissure, was identified, and two perpendicular axes were drawn to measure the lesion. These measurements were also performed on coronal slices along the ventrodorsal axis, and the product of three measurements (A, B, and C) was calculated, then divided by two to estimate the volume in cubic centimeters. Second, 3D multiplanar segmentation was conducted using Image Tool Kit-SNAP (ITK-SNAP) software (version 3.8.0, University of Pennsylvania) [20]. Sequential image stitching was performed to include the maximum number of slices and sequences. The tumor and edema were manually delineated on the contrast-enhanced T1 and T2 sequences, respectively, with the software automatically calculating the volumetric measurements in cubic centimeters [21,22].

ETI calculation

ETI was calculated by dividing the volume of edema by the volume of the tumor, expressed as an inverse ratio. This index aimed to provide a quantitative measure of the relationship between tumor size and the amount of peritumoral edema, which may have prognostic implications for malignancy.

Statistical analysis

All collected data, including sociodemographic information and volumetric measurements, were compiled and analyzed using Statistical Package for the Social Sciences (SPSS) software (version 21, IBM Corp., Armonk, NY). A comparison of means was performed using the T-student test for independent samples to analyze differences in tumor and edema volumes. Receiver Operating Characteristic (ROC) curve analysis was conducted to establish cutoff points for the ETI, with the area under the curve (AUC) and Youden index used to evaluate diagnostic accuracy.

Data management and ethical considerations

All imaging studies were stored and organized in PowerPoint presentations for analysis, and these files were subsequently deleted after the study's completion to ensure data security. Patient confidentiality was maintained by ethical standards and regulations. The study was conducted following ethical guidelines, ensuring all collected data were anonymized, and no patient-identifying information was used.

## Results

**Figure 1:**
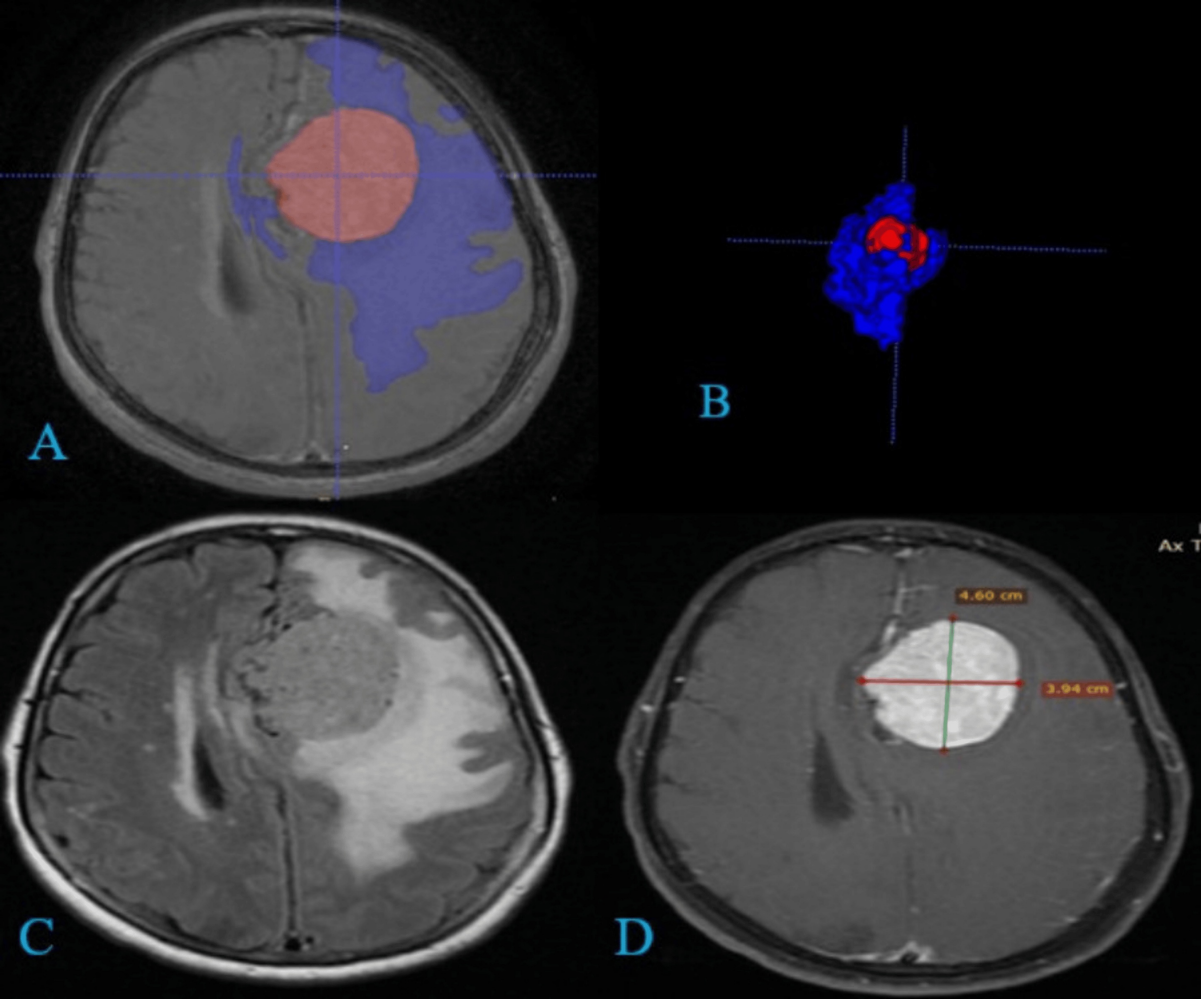
(A and B) 3D segmentation with volumetry of a meningioma (grade 1) of the cerebral falx. Definitive diagnosis: WHO grade 1 transitional meningioma. Tumor volume: 45 cc. Peritumoral edema volume: 147 cc. Edema/tumor index: 3.2. (C and D) Same tumor with conventional volumetry (AUC/2). Tumor volume: 39.7 cc (volumetric underestimation). Edema volume: 220 cc, estimated using the formula: (V_edema_ + V_tumor_)/V_tumor_) (volumetric overestimation). AUC, area under the curve

**Figure 2:**
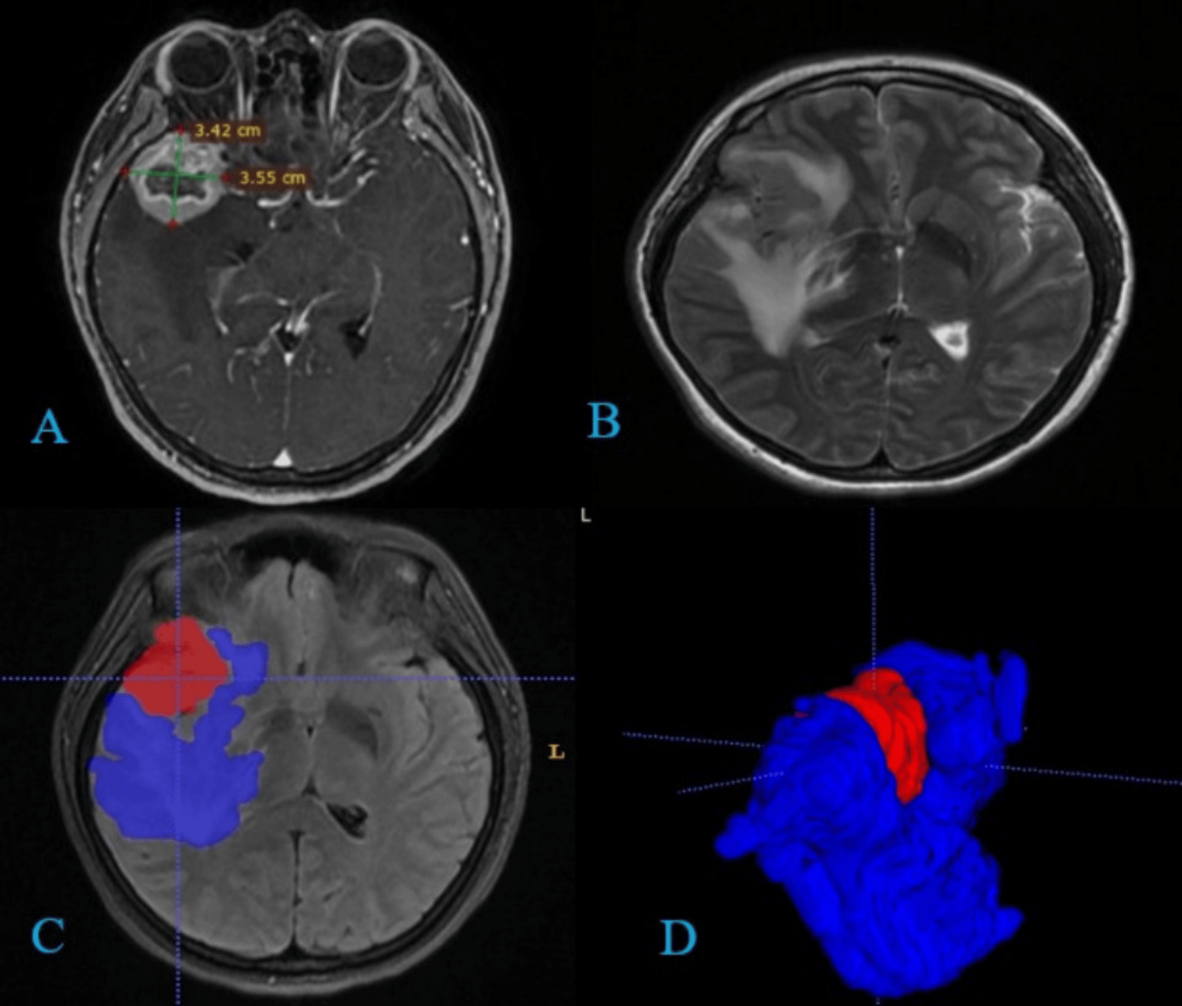
(A and B) Axial sections in contrasted T1 and T2 sequences, where conventional uniplanar volumetry of a metastatic breast lesion was performed. (C and D) Manual 3D volumetry with tumor areas delimited in red and edema in blue. Tumor volume in 2D image, 22.4 cc. 3D volume, tumor 25 cc, edema 90.8 cc, and edema/tumor ratio: 3.6.

A retrospective analysis was conducted on 60 patients, comprising 27 men (45%) and 33 women (55%). The mean age was 55 years, with an interquartile range of 55 years (50%). Twenty-six tumors were classified as malignant (defined as WHO grades 3 and 4), accounting for 43.3% of the cases, while 34 were non-malignant (WHO grades 1 and 2), representing 56.7% of the cases. The mean tumor size measured by 2D planimetric sequence was 42.6 cubic centimeters (cc) (SD ± 25.6), whereas the mean tumor size measured by three-dimensional manual segmentation was 46.9 cc (SD ± 25.6). A Pearson correlation test yielded a value of 0.936, indicating a strong correlation between both measurement methods (*P* < 0.05) (Figures 1-2).

No correlation test was conducted for edema since it was not calculated in a 2D manner. An independent samples t-test was performed to evaluate the equality of means by sex and tumor size. In males, the mean tumor volume was 49.9 cc, and in females, it was 44.5 cc, with SDs of ±28.9 and ±22.8 cc, respectively, showing no significant difference (*P* = 0.320). The same test was conducted to determine the means in malignant and non-malignant tumors. The mean volume for malignant tumors was 61 cc (SD ±26.7), while for non-malignant tumors, it was 35.7 cc (SD ±18.3), a statistically significant difference (*P* = 0.03). Furthermore, the variance equality analysis revealed a mean difference of 25 cc, with a 95% confidence interval (CI) of 14-37 cc, which was statistically significant (*P* < 0.05). This indicates that histologically malignant tumors have a 25 cc greater volume than non-malignant ones.

**Figure 3:**
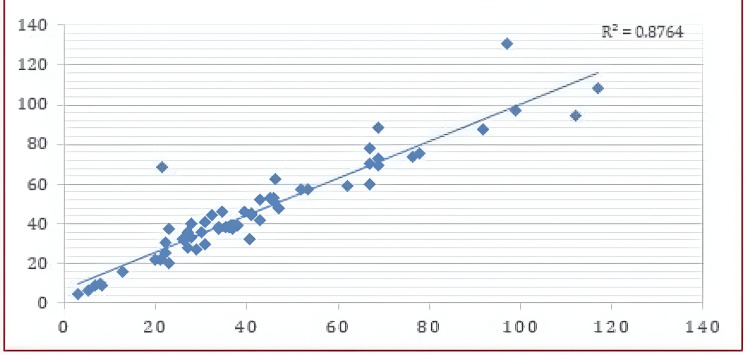
Linear correlation between both measurements, both by conventional 2D uniplanar image and 3D by segmentation. X-axis: Tumor volume (cubic centimeters, cc). Y-axis: Edema volume (cubic centimeters, cc).

Regarding the edema generated by the tumors, the same test was conducted, showing a mean edema volume of 79 cc for malignant tumors compared to 42 cc for non-malignant ones, with a significant mean difference of 37 cc (*P* < 0.05). Finally, the ETI was calculated. The mean index for malignant tumors was 1.6 (SD ±1.2), while for non-malignant tumors, it was 1.2 (SD ±1.1), which was not statistically significant (*P* = 0.51) (Figure 3).

**Figure 4:**
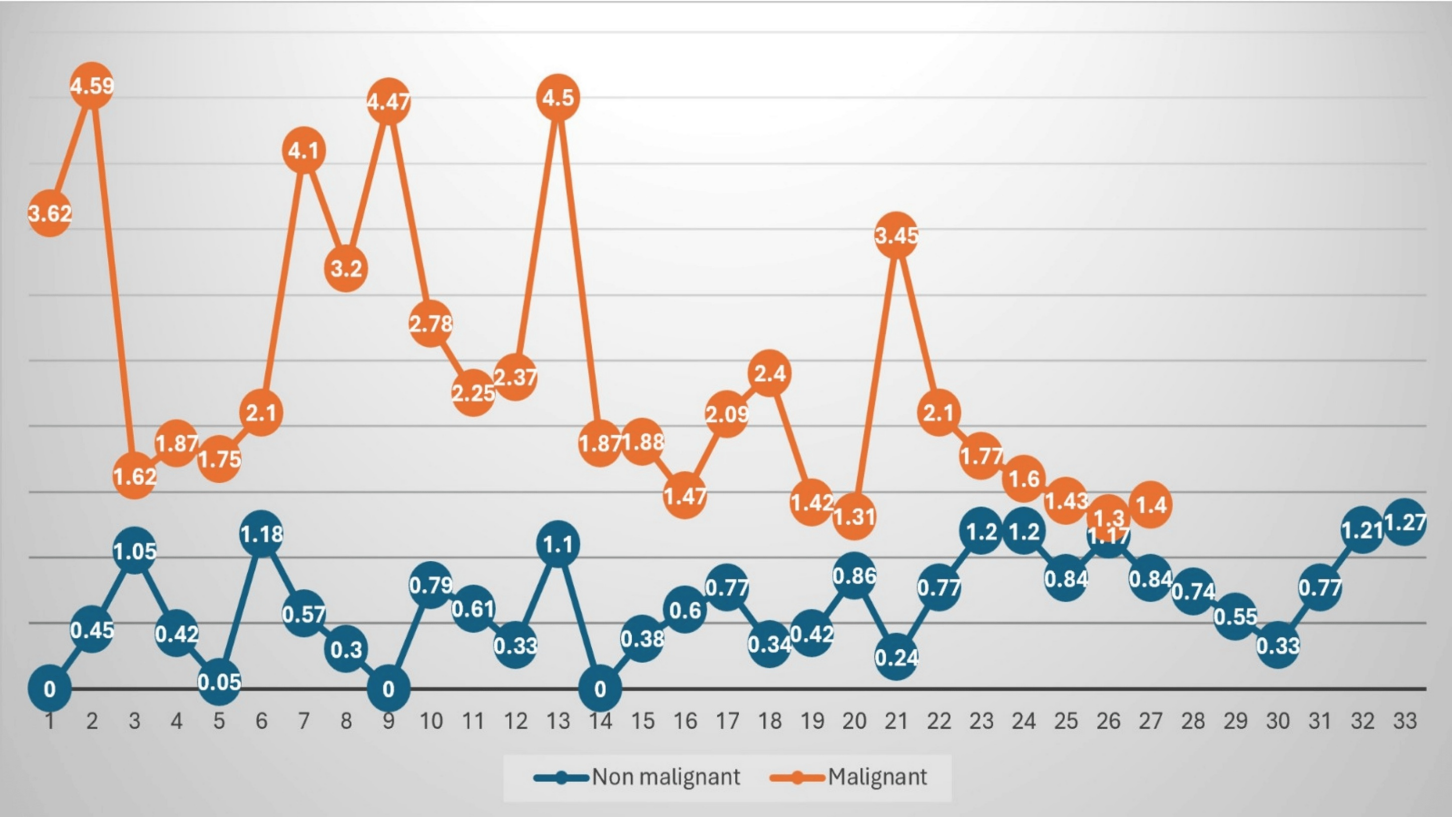
Comparative graph of edema/tumor ratio in the 60 tumors measured by 3D segmentation. There is a marked tendency to have a higher rate in malignant tumors (orange) than in non-malignant tumors (blue).

The AUC was calculated using ROC curves, yielding an AUC of 0.59 (95% CI 0.49-0.74), with *P* = 0.20. The Youden index was used to establish a cutoff point, set at 1.3, with a 1-specificity value of 0.38 and a sensitivity of 0.53. Based on this cutoff point, the population was dichotomized into those with an ETI below 1.3 and those above this value. A total of 33 patients (55%) were below the threshold, and 27 patients (45%) were above the established ETI (Figure 4). A Chi-square test yielded a Chi-square value of 1.41 and a *P*-value of 0.17, indicating no significant association between an ETI of 1.3 or greater and tumor malignancy.

## Discussion

In this study, we evaluated the correlation between the ETI and the histopathological grade of brain tumors, as classified by the 2021 WHO guidelines. Our results showed that malignant tumors (WHO grades 3 and 4) were associated with larger volumes of both tumor and peritumoral edema when compared to non-malignant tumors (WHO grades 1 and 2). However, despite these volumetric differences, the ETI did not demonstrate a statistically significant correlation with tumor malignancy. ROC curve analysis also failed to establish a reliable cut-off point for ETI predictive of higher tumor grades. Thus, the ETI, in its current form, does not appear to be a reliable predictor of tumor aggressiveness.

When comparing our findings to existing literature, some parallels and discrepancies emerge. Yushkevich et al. [10] and Latini et al. [23] demonstrated the superiority of 3D manual segmentation for volumetric accuracy, which aligns with our decision to use ITK-SNAP for tumor and edema volume measurement. This segmentation approach has consistently been regarded as the gold standard for volumetric analysis, and our results further support its efficacy in measuring complex tumor shapes and associated edema.

Hanum et al. [7] and Bečulić et al. [24] explored the edema/tumor volume ratio and its predictive value for tumor malignancy, finding stronger correlations between the ratio and tumor aggressiveness than we observed. While these studies support the use of the edema/tumor ratio as a diagnostic tool, our study found no significant correlation between the ETI and histopathological grade. One potential explanation for this discrepancy could be the relatively small sample size of 60 patients in our cohort, which may have limited the statistical power of our analysis.

Furthermore, Xiao et al. [11] used volumetric measurements to differentiate between necrotic glioblastomas and brain abscesses, demonstrating that volumetric indices, such as the edema/tumor ratio, can have diagnostic utility. Although we employed a similar volumetric approach, our results suggest that ETI may not be sufficient as a standalone marker of malignancy in brain tumors. This highlights the need for further refinement of volumetric indices and their integration with other imaging and molecular markers to enhance diagnostic accuracy.

Chang et al. [22] emphasized the role of tumor size in predicting malignancy and clinical outcomes. Consistent with their findings, we observed that larger tumor volumes were associated with higher tumor grades in our cohort. However, our study did not find a significant relationship between ETI and malignancy, suggesting that factors other than edema volume may play a more critical role in determining tumor aggressiveness.

Tumor-induced edema is often linked with higher malignancy grades and a poorer prognosis for survival. Interestingly, it is not only malignant tumors that can cause edema. Benign meningiomas, which constitute approximately 92% of cases, also have the potential to produce endothelial growth factors and pro-inflammatory cytokines that contribute to peritumoral edema formation. Although the precise mechanisms through which tumors, including meningiomas, generate edema are not fully understood, significant efforts have been made to quantify this edema using volumetric imaging techniques [18,19].

Traditional measurement approaches, such as the *edema index* ([V_edema_ + V_tumor_]/V_tumor_), rely on spherical models and provide approximate results due to the inherently irregular shape of edema. Nonetheless, these methods have been instrumental in large-scale studies that examine the relationship between edema and survival outcomes [20,21]. A notable study conducted by Bečuli in 2019 involving 41 patients with intracranial meningioma identified the peritumoral edema index as a novel predictor of mortality [22]. This study employed manual segmentation for volumetric analysis, which is widely regarded as the gold standard for accuracy.

Further research has highlighted the complexity of edema in various tumor types. Advanced imaging modalities, such as MRI and CT scans, play a crucial role in assessing the extent of peritumoral edema and its impact on patient outcomes (Figures 1-2). These imaging techniques, combined with sophisticated software for 3D reconstruction and volumetric analysis, have enhanced our ability to evaluate and monitor tumor-associated edema [23-25].

In clinical practice, understanding the dynamics of peritumoral edema is essential for tailoring treatment strategies. For example, in the management of meningiomas, preoperative assessment of edema can influence surgical planning and postoperative care. The administration of corticosteroids to reduce edema and improve neurological function is a common therapeutic approach, highlighting the importance of accurate edema measurement in guiding clinical decisions [26-28].

Continued advancements in imaging technology and computational analysis are expected to refine our understanding of tumor-generated edema. As we gain deeper insights into the biological mechanisms driving edema formation, new therapeutic targets may emerge, potentially leading to improved patient outcomes across various tumor types [29,30].

A similar study by Vijay et al. compared the mean ETI between primary tumors and metastases. In this study, segmentation was performed automatically using a 3D viewer with contrast-enhanced T1 and T2 FLAIR sequences [31]. While this automated method offers efficiency, it has limitations: edema zones not identified by the software may persist, making manual segmentation, which leverages anatomical knowledge, more accurate [32].

The software used, ITK-SNAP, can automatically provide the volume of manually marked structures by calculating voxels within the segmentation (planimetry). This free-access software was referenced in a 2021 study by Xiao et al., which used images from 678 patients with meningiomas to compare various conventional volumetric methods (1/3ABC, 1/2ABC, 1/3SH, 1/2SH, among others) for tumor volume measurement [33]. These methods were evaluated against the gold standard of 3D planimetric segmentation. The findings revealed that the 1/3ABC method tends to overestimate volume, while the 1/3SH method tends to underestimate it. Interestingly, these are the methods most frequently used in daily practice [34].

Another important application of segmentation is to assist in the definitive diagnosis of tumor lesions or lesions mimicking tumors, such as brain abscesses and necrotic gliomas [35,36]. High-performance models use the ETI calculated by segmentation to discriminate between tumor lesions (necrotic gliomas) and brain abscesses, demonstrating good reproducibility results [37].

Tumor size is a crucial characteristic, as larger tumors are often associated with more symptoms, complications, and treatment challenges. While larger tumor size has not been definitively proven to increase the probability of malignancy due to the heterogeneity of brain tumors, there is a recognized relationship between size and prognosis [38,39]. Larger tumors tend to generate more edema, which can complicate clinical management [28,30].

In the context of benign tumors like meningiomas, edema is related to factors such as the proliferation index, pial invasion, and long-term recurrence. There is also a notable association between the degree of edema and the Ki-67 proliferation index in glial tumors, with a higher correlation observed in highly malignant glial tumors. Although no statistically significant association was found in our study, there was a tendency toward higher edema indices in malignant tumors [17,34,38]. This may be attributed to the larger tumor size and the greater volume of edema generated by more aggressive malignancies.

Tumor-generated edema, often referred to as vasogenic edema, arises due to the disruption of the blood-brain barrier (BBB) and subsequent leakage of plasma into the extracellular space [39]. This phenomenon is prevalent in both malignant and benign tumors, albeit with varying intensity and patterns. For instance, high-grade gliomas exhibit pronounced vasogenic edema due to their aggressive nature and angiogenic activity, whereas benign tumors like meningiomas can also produce significant edema through the secretion of vascular endothelial growth factors (VEGF) and other pro-inflammatory cytokines [40,41].

The accurate measurement of edema and tumor volumes remains a critical challenge. Traditional uniplanar imaging methods, relying on geometric assumptions, often yield approximate results due to the irregular shape of both tumors and the associated edema [42,43]. The adoption of manual 3D segmentation, as employed in this study, offers a more precise volumetric assessment. However, it is labor-intensive and requires significant expertise, limiting its widespread applicability in routine clinical practice [44,45].

While the study showed no significant correlation between ETI and malignancy grades, previous research suggests potential prognostic value. For example, a 2016 study by Sunderland et al., highlighted the ETI as a predictor of survival in patients with posterior fossa metastases, emphasizing the need for further validation in diverse tumor types and larger cohorts [46]. The lack of significant findings in this study could be attributed to the small sample size and the inherent variability in tumor biology [47].

Future directions

The incorporation of artificial intelligence (AI) and machine learning (ML) in the analysis of the ETI represents a transformative approach with the potential to significantly enhance diagnostic precision, prognostic accuracy, and treatment planning in neuro-oncology [48].

Automated 3D Segmentation

AI-driven automated segmentation tools can drastically reduce the time and effort required for manual 3D segmentation. Machine learning algorithms, particularly deep learning models, can be trained on large datasets to accurately delineate tumor and edema regions [49,50]. These models can learn complex patterns and variations in imaging data, providing consistent and reproducible volumetric measurements. Recent advancements in convolutional neural networks (CNNs) and other deep learning architectures have shown promising results in medical image segmentation, outperforming traditional methods in both accuracy and efficiency [51-53].

Enhanced Predictive Modeling

Machine learning algorithms can analyze large and complex datasets to identify subtle patterns and correlations that may not be evident through conventional statistical methods. By incorporating a wide array of clinical, imaging, and molecular data, AI can develop predictive models that assess the likelihood of tumor malignancy, patient prognosis, and response to treatment based on ETI and other relevant factors. These models can continuously improve as they are exposed to more data, enhancing their predictive power over time [54,55].

Radiomics and Radiogenomics

Radiomics involves extracting a large number of quantitative features from medical images, which can then be analyzed using machine learning techniques. Radiogenomics extends this approach by linking imaging features with genomic and molecular data [56]. This integration can provide a more comprehensive understanding of tumor biology and its relationship with imaging characteristics, including the ETI. AI algorithms can analyze radiomic and radiogenomic features to develop biomarkers that predict tumor behavior, treatment response, and clinical outcomes [57].

Personalized Treatment Planning

AI can assist in developing personalized treatment plans by analyzing individual patient data and predicting the most effective therapeutic strategies. For instance, machine learning models can predict which patients are likely to benefit from specific treatments based on their ETI and other clinical parameters. This personalized approach can optimize treatment efficacy, minimize adverse effects, and improve overall patient outcomes [58].

Real-Time Decision Support

AI-powered decision support systems can provide real-time analysis and recommendations during clinical workflows. These systems can alert clinicians to significant changes in ETI or other critical parameters, enabling timely interventions. For example, an AI system could monitor serial imaging studies to detect early signs of treatment response or disease progression, allowing for rapid adjustments in the therapeutic approach.

Integration With Electronic Health Records (EHRs)

Integrating AI algorithms with EHRs can facilitate comprehensive data analysis, encompassing clinical history, imaging studies, laboratory results, and molecular profiles. This holistic view can enhance the accuracy of ETI-based prognostic models and support informed clinical decision-making. Moreover, AI can streamline data management and analysis, reducing the administrative burden on healthcare providers [59,60-62].

Continuous Learning and Improvement

AI systems can continuously learn and adapt as they process more data, improving their performance over time. This capability is particularly valuable in the dynamic field of neuro-oncology, where new research findings and clinical insights are constantly emerging. Machine learning models can be updated regularly with the latest data, ensuring that they remain current and effective in guiding clinical practice [63-65].

Collaborative Research and Data Sharing

The development of AI and machine learning models requires access to large, diverse datasets. Collaborative research initiatives and data-sharing platforms can facilitate the pooling of imaging and clinical data from multiple institutions. This collective approach can enhance the robustness and generalizability of AI algorithms, ensuring that they are applicable across different patient populations and healthcare settings [66,67].

Limitations of the study

Given the study’s focus on anatomical and volumetric data, this approach may not capture molecular or genetic variations that can impact tumor behavior.

While this study provides valuable insights into the correlation between tumor volume and edema using both 3D volumetric segmentation and conventional 2D imaging techniques, several limitations must be acknowledged. First, the relatively small sample size of 60 patients limits the generalizability of our findings. A larger cohort would enhance the statistical power and allow for more robust conclusions, particularly regarding the ETI and its prognostic value.

The study relied on manual 3D segmentation, which, while more accurate than geometric methods, is time-consuming and subject to variability based on the experience of the operator. Future studies incorporating automated or semi-automated segmentation methods could reduce bias and improve reproducibility.

Additionally, the retrospective nature of the study limits the ability to control for potential confounding variables, such as variations in tumor location, patient treatment histories, and pre-existing conditions, which may influence the extent of edema.

While the study demonstrated significant differences in tumor and edema volumes between high-grade and non-malignant tumors, the absence of a significant correlation between ETI and malignancy suggests that other factors, such as tumor biology or molecular characteristics, may play a larger role in determining edema volume. Further research with larger samples and advanced molecular analyses is needed to refine our understanding of these relationships.

## Conclusions

This study shows that malignant lesions have a larger tumor size compared to benign lesions. A linear correlation was found between the volumetric measurement methods (planar and 3D segmentation), as reported in some studies. The edema/tumor index did not correlate with the degree of malignancy, nor was there any association between tumor size, edema size, or sex. This study could not establish a cut-off point for predicting tumor prognosis based on imaging studies. Therefore, volumetric measurement techniques should not be used as a basis for decision-making at this time. Despite the study's limitations due to the small sample size, we can conclude that uniplanar 2D measurement remains a quick, effective, and accurate strategy for approximating the real total volume. Statistically significant differences were found in the amount of tumor and edema tissue between high-grade and non-malignant tumors. However, there is no valid argument to suspect malignant tumors based on higher edema volume, as no correlation was found between higher edema/tumor indices and WHO grade 3 or 4 tumors.
